# Biphenyl-4,4′-diyl bis­(2,2,5,5-tetra­methyl-1-oxyl-3-pyrroline-3-carboxyl­ate)

**DOI:** 10.1107/S1600536809024659

**Published:** 2009-07-04

**Authors:** Dominik Margraf, Denise Schuetz, Thomas F. Prisner, Jan W. Bats

**Affiliations:** a Institut für Physikalische und Theoretische Chemie, Universität Frankfurt, Max-von-Laue-Strasse 7, D-60438 Frankfurt am Main, Germany; b Institut für Organische Chemie, Universität Frankfurt, Max-von-Laue-Strasse 7, D-60438 Frankfurt am Main, Germany

## Abstract

In the title compound, C_30_H_34_N_2_O_6_, the complete molecule is generated by a crystallographic 2/*m* symmetry operation. The 1-oxyl-3-pyrroline-3-carboxyl­ate group lies on a mirror plane. The dihedral angle between the ring planes of the biphenyl fragment is constrained by symmetry to be zero, resulting in rather short intramolecular H⋯H contact distances of 2.02 Å. In the crystal, molecules are connected along the *a*-axis direction by very weak intermolecular methyl–phenyl C—H⋯π interactions. The C—H bond is not directed to the center of the benzene ring, but mainly to one C atom [C—H⋯C(*x* − 1, *y*, *z*): H⋯C = 2.91 Å and C—H⋯C = 143°].

## Related literature

For the preparation of the title compound see: Weber *et al.* (2002 ▶). For the crystal structures of related compounds see: Boeyens & Kruger (1970 ▶); Bolte (2006 ▶); Duskova *et al.* (2001 ▶); Godt *et al.*, 2000 ▶; Papoutsakis *et al.* (1999 ▶); Wiley *et al.*, 1989 ▶ and Wiley *et al.*, 1991 ▶.
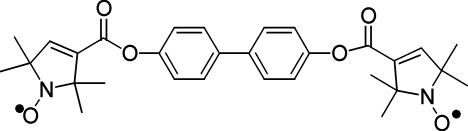


## Experimental

### 

#### Crystal data

C_30_H_34_N_2_O_6_
*M* *_r_* = 518.59
Monoclinic, 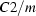

*a* = 6.931 (2) Å
*b* = 9.461 (3) Å
*c* = 20.805 (4) Å
β = 96.059 (14)°
*V* = 1356.6 (6) Å^3^
*Z* = 2
Mo *K*α radiation
μ = 0.09 mm^−1^
*T* = 169 K
0.44 × 0.30 × 0.10 mm

#### Data collection

Siemens SMART 1K CCD diffractometer
Absorption correction: none
11267 measured reflections
2074 independent reflections
1552 reflections with *I* > 2σ(*I*)
*R*_int_ = 0.056

#### Refinement

*R*[*F* ^2^ > 2σ(*F* ^2^)] = 0.049
*wR*(*F* ^2^) = 0.126
*S* = 1.03
2074 reflections
105 parameters
H-atom parameters constrained
Δρ_max_ = 0.39 e Å^−3^
Δρ_min_ = −0.21 e Å^−3^

### 

Data collection: *SMART* (Siemens, 1995 ▶); cell refinement: *SMART*; data reduction: *SAINT* (Siemens, 1995 ▶); program(s) used to solve structure: *SHELXS97* (Sheldrick, 2008 ▶); program(s) used to refine structure: *SHELXL97* (Sheldrick, 2008 ▶); molecular graphics: *SHELXTL* (Sheldrick, 2008 ▶); software used to prepare material for publication: *SHELXL97*.

## Supplementary Material

Crystal structure: contains datablocks global, I. DOI: 10.1107/S1600536809024659/lh2852sup1.cif

Structure factors: contains datablocks I. DOI: 10.1107/S1600536809024659/lh2852Isup2.hkl

Additional supplementary materials:  crystallographic information; 3D view; checkCIF report

## Figures and Tables

**Table 1:** Hydrogen-bond geometry (Å, °)

*D*—H⋯*A*	*D*—H	H⋯*A*	*D*⋯*A*	*D*—H⋯*A*
C11—H11*C*⋯C3^i^	0.98	2.91	3.745 (2)	143

Symmetry code: (i) 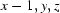.

**Fig. 1.**
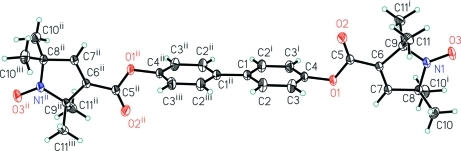
The structure of the title compound shown with 50% probability displacement ellipsoids. The H atoms are drawn as small spheres of arbitrary radius. Symmetry equivalent atoms are related by i: x, -y, z, ii: 2 - x, -y, 1 - z and iii: 2 - x, y, 1 - z.

**Fig. 2.**
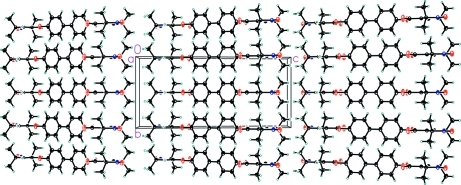
The crystal packing of the title compound, viewed down the a axis.

## supplementary crystallographic information

### Comment

The title compound was prepared as a reference compound for pulsed electron-electron double resonance measurements (Weber *et al.*, 2002).

The molecular structure is shown in Fig. 1. The molecule has 2/m symmetry: atoms C1, C4, O1, C5, O2, C6, C7, C8, C9, N1 and O3 lie on a mirror plane. There is a twofold axis perpendicular to this mirror plane and passing through the center of the central C—C single bond. There also is an inversion center at the midpoint of the central C—C single bond. The two six-membered rings of the biphenyl group are coplanar by symmetry, resulting in rather short intramolecular H···H contact distances of 2.02 A. The 1-oxyl-3-pyrroline-3-carboxylate group is planar. Approximate planarity of this group also has been observed in a number of related crystal structures (Papoutsakis *et al.*, 1999; Boeyens & Kruger, 1970; Bolte, 2006; Duskova *et al.*, 2001; Godt *et al.*, 2000; Wiley *et al.*, 1989 and Wiley *et al.*, 1991)

The crystal packing is shown in Fig 2. The molecules are connected along the a-direction by four symmetry-equivalent very weak intermolecular C_methyl_—H···π(phenyl) interactions (Table 1). The C_methyl_—H bond is not directed to the center of the phenyl ring, but mainly to one C atom. There are no other short intermolecular contacts.

### Experimental

The title compound was prepared similar to the procedure described by Weber *et al.* (2002). Single crystals were obtained by recrystallization of the compound from a mixture of toluene and n-hexane (3:1).

### Refinement

The H atoms were positioned geometrically and treated as riding: C_methyl_—H=0.98 Å, C_planar_—H=0.95 Å, *U*_iso_(*H*)=1.2*U*_eq_(C_non-methyl_) and *U*_iso_(*H*)=1.5*U*_eq_(C_methyl_). The torsion angles about the *C*—C_methyl_ bonds were refined for the methyl groups.

### Crystal data

**Table:**

C_30_H_34_N_2_O_6_	*F*(000) = 552
*M**_r_* = 518.59	*D*_x_ = 1.270 Mg m^−^^3^
Monoclinic, *C*2/*m*	Mo *K*α radiation, λ = 0.71073 Å
Hall symbol: -C 2yc	Cell parameters from 77 reflections
*a* = 6.931 (2) Å	θ = 3–23°
*b* = 9.461 (3) Å	µ = 0.09 mm^−^^1^
*c* = 20.805 (4) Å	*T* = 169 K
β = 96.059 (14)°	Plate, yellow
*V* = 1356.6 (6) Å^3^	0.44 × 0.30 × 0.10 mm
*Z* = 2	

### Data collection

**Table:**

Siemens SMART 1K CCD diffractometer	1552 reflections with *I* > 2σ(*I*)
Radiation source: normal-focus sealed tube	*R*_int_ = 0.056
graphite	θ_max_ = 30.5°, θ_min_ = 2.0°
ω scans	*h* = −9→9
11267 measured reflections	*k* = −13→12
2074 independent reflections	*l* = −29→29

### Refinement

**Table:**

Refinement on *F*^2^	Primary atom site location: structure-invariant direct methods
Least-squares matrix: full	Secondary atom site location: difference Fourier map
*R*[*F*^2^ > 2σ(*F*^2^)] = 0.049	Hydrogen site location: inferred from neighbouring sites
*wR*(*F*^2^) = 0.126	H-atom parameters constrained
*S* = 1.03	*w* = 1/[σ^2^(*F*_o_^2^) + (0.06*P*)^2^ + 0.9*P*] where *P* = (*F*_o_^2^ + 2*F*_c_^2^)/3
2074 reflections	(Δ/σ)_max_ = 0.001
105 parameters	Δρ_max_ = 0.39 e Å^−^^3^
0 restraints	Δρ_min_ = −0.21 e Å^−^^3^

### Special details

**Table:**

Geometry. All e.s.d.'s (except the e.s.d. in the dihedral angle between two l.s. planes) are estimated using the full covariance matrix. The cell e.s.d.'s are taken into account individually in the estimation of e.s.d.'s in distances, angles and torsion angles; correlations between e.s.d.'s in cell parameters are only used when they are defined by crystal symmetry. An approximate (isotropic) treatment of cell e.s.d.'s is used for estimating e.s.d.'s involving l.s. planes.
Refinement. Refinement of *F*^2^ against ALL reflections. The weighted *R*-factor *wR* and goodness of fit *S* are based on *F*^2^, conventional *R*-factors *R* are based on *F*, with *F* set to zero for negative *F*^2^. The threshold expression of *F*^2^ > σ(*F*^2^) is used only for calculating *R*-factors(gt) *etc*. and is not relevant to the choice of reflections for refinement. *R*-factors based on *F*^2^ are statistically about twice as large as those based on *F*, and *R*- factors based on ALL data will be even larger.

### Fractional atomic coordinates and isotropic or equivalent isotropic displacement parameters (Å^2^)

**Table:**

	*x*	*y*	*z*	*U*_iso_*/*U*_eq_	
O1	0.58234 (19)	0.0000	0.29187 (6)	0.0310 (3)	
O2	0.2989 (2)	0.0000	0.33551 (6)	0.0396 (4)	
O3	−0.0951 (2)	0.0000	0.09831 (7)	0.0323 (3)	
N1	0.0672 (2)	0.0000	0.13356 (7)	0.0231 (3)	
C1	0.9329 (3)	0.0000	0.46943 (8)	0.0231 (4)	
C2	0.8678 (2)	0.12604 (16)	0.43958 (6)	0.0317 (3)	
H2A	0.9076	0.2135	0.4592	0.038*	
C3	0.7462 (2)	0.12691 (16)	0.38187 (7)	0.0321 (3)	
H3A	0.7036	0.2137	0.3622	0.039*	
C4	0.6887 (3)	0.0000	0.35376 (8)	0.0261 (4)	
C5	0.3862 (3)	0.0000	0.28870 (8)	0.0211 (4)	
C6	0.2971 (2)	0.0000	0.22093 (8)	0.0179 (3)	
C7	0.3911 (3)	0.0000	0.16826 (8)	0.0190 (3)	
H7A	0.5285	0.0000	0.1696	0.023*	
C8	0.2570 (3)	0.0000	0.10658 (8)	0.0205 (3)	
C9	0.0794 (2)	0.0000	0.20539 (8)	0.0185 (3)	
C10	0.2784 (2)	0.13302 (16)	0.06612 (7)	0.0324 (3)	
H10A	0.1799	0.1325	0.0287	0.049*	
H10B	0.4079	0.1348	0.0512	0.049*	
H10C	0.2611	0.2169	0.0925	0.049*	
C11	−0.01778 (19)	0.13337 (14)	0.22809 (7)	0.0261 (3)	
H11A	−0.1535	0.1360	0.2093	0.039*	
H11B	0.0503	0.2170	0.2142	0.039*	
H11C	−0.0125	0.1327	0.2753	0.039*	

### Atomic displacement parameters (Å^2^)

**Table:**

	*U*^11^	*U*^22^	*U*^33^	*U*^12^	*U*^13^	*U*^23^
O1	0.0170 (6)	0.0590 (9)	0.0159 (6)	0.000	−0.0032 (5)	0.000
O2	0.0240 (7)	0.0753 (12)	0.0194 (6)	0.000	0.0022 (5)	0.000
O3	0.0215 (7)	0.0429 (8)	0.0295 (7)	0.000	−0.0116 (5)	0.000
N1	0.0176 (7)	0.0302 (8)	0.0200 (7)	0.000	−0.0050 (5)	0.000
C1	0.0194 (8)	0.0309 (9)	0.0182 (8)	0.000	−0.0018 (6)	0.000
C2	0.0363 (8)	0.0306 (7)	0.0256 (7)	0.0027 (6)	−0.0087 (6)	−0.0022 (5)
C3	0.0340 (8)	0.0360 (8)	0.0244 (6)	0.0067 (6)	−0.0066 (5)	0.0026 (6)
C4	0.0166 (8)	0.0452 (11)	0.0157 (8)	0.000	−0.0023 (6)	0.000
C5	0.0183 (8)	0.0246 (8)	0.0195 (8)	0.000	−0.0017 (6)	0.000
C6	0.0164 (7)	0.0172 (7)	0.0192 (8)	0.000	−0.0020 (6)	0.000
C7	0.0179 (8)	0.0188 (8)	0.0193 (8)	0.000	−0.0021 (6)	0.000
C8	0.0211 (8)	0.0226 (8)	0.0169 (7)	0.000	−0.0015 (6)	0.000
C9	0.0159 (7)	0.0193 (8)	0.0199 (8)	0.000	−0.0006 (6)	0.000
C10	0.0376 (8)	0.0321 (7)	0.0262 (7)	−0.0044 (6)	−0.0032 (6)	0.0093 (6)
C11	0.0200 (6)	0.0234 (6)	0.0346 (7)	0.0024 (5)	0.0012 (5)	−0.0035 (5)

### Geometric parameters (Å, °)

**Table:**

O1—C5	1.354 (2)	C6—C7	1.332 (2)
O1—C4	1.414 (2)	C6—C9	1.509 (2)
O2—C5	1.199 (2)	C7—C8	1.503 (2)
O3—N1	1.2766 (19)	C7—H7A	0.9500
N1—C8	1.484 (2)	C8—C10^i^	1.5299 (17)
N1—C9	1.488 (2)	C8—C10	1.5299 (17)
C1—C2^i^	1.3970 (17)	C9—C11	1.5285 (16)
C1—C2	1.3971 (17)	C9—C11^i^	1.5285 (16)
C1—C1^ii^	1.495 (3)	C10—H10A	0.9800
C2—C3	1.3922 (19)	C10—H10B	0.9800
C2—H2A	0.9500	C10—H10C	0.9800
C3—C4	1.3760 (17)	C11—H11A	0.9800
C3—H3A	0.9500	C11—H11B	0.9800
C4—C3^i^	1.3761 (17)	C11—H11C	0.9800
C5—C6	1.478 (2)		
			
C5—O1—C4	117.92 (14)	N1—C8—C7	99.78 (13)
O3—N1—C8	123.07 (14)	N1—C8—C10^i^	110.45 (10)
O3—N1—C9	122.02 (15)	C7—C8—C10^i^	112.51 (9)
C8—N1—C9	114.91 (13)	N1—C8—C10	110.45 (10)
C2^i^—C1—C2	117.19 (16)	C7—C8—C10	112.51 (9)
C2^i^—C1—C1^ii^	121.40 (8)	C10^i^—C8—C10	110.69 (15)
C2—C1—C1^ii^	121.40 (8)	N1—C9—C6	99.50 (13)
C3—C2—C1	121.74 (13)	N1—C9—C11	109.27 (9)
C3—C2—H2A	119.1	C6—C9—C11	113.40 (9)
C1—C2—H2A	119.1	N1—C9—C11^i^	109.28 (9)
C4—C3—C2	118.90 (13)	C6—C9—C11^i^	113.40 (9)
C4—C3—H3A	120.6	C11—C9—C11^i^	111.28 (15)
C2—C3—H3A	120.6	C8—C10—H10A	109.5
C3—C4—C3^i^	121.52 (17)	C8—C10—H10B	109.5
C3—C4—O1	119.12 (8)	H10A—C10—H10B	109.5
C3^i^—C4—O1	119.12 (8)	C8—C10—H10C	109.5
O2—C5—O1	123.36 (16)	H10A—C10—H10C	109.5
O2—C5—C6	125.39 (17)	H10B—C10—H10C	109.5
O1—C5—C6	111.25 (14)	C9—C11—H11A	109.5
C7—C6—C5	126.39 (16)	C9—C11—H11B	109.5
C7—C6—C9	112.83 (14)	H11A—C11—H11B	109.5
C5—C6—C9	120.78 (14)	C9—C11—H11C	109.5
C6—C7—C8	112.99 (16)	H11A—C11—H11C	109.5
C6—C7—H7A	123.5	H11B—C11—H11C	109.5
C8—C7—H7A	123.5		
			
C2^i^—C1—C2—C3	−1.2 (3)	C9—N1—C8—C10^i^	118.61 (10)
C1^ii^—C1—C2—C3	178.55 (19)	O3—N1—C8—C10	61.39 (10)
C1—C2—C3—C4	0.1 (2)	C9—N1—C8—C10	−118.61 (10)
C2—C3—C4—C3^i^	1.0 (3)	C6—C7—C8—N1	0.0
C2—C3—C4—O1	−173.34 (14)	C6—C7—C8—C10^i^	−117.08 (11)
C5—O1—C4—C3	−92.77 (15)	C6—C7—C8—C10	117.08 (11)
C5—O1—C4—C3^i^	92.77 (15)	O3—N1—C9—C6	180.0
C4—O1—C5—O2	0.0	C8—N1—C9—C6	0.0
C4—O1—C5—C6	180.0	O3—N1—C9—C11	−60.99 (10)
O2—C5—C6—C7	180.0	C8—N1—C9—C11	119.01 (10)
O1—C5—C6—C7	0.0	O3—N1—C9—C11^i^	60.99 (10)
O2—C5—C6—C9	0.0	C8—N1—C9—C11^i^	−119.01 (10)
O1—C5—C6—C9	180.0	C7—C6—C9—N1	0.0
C5—C6—C7—C8	180.0	C5—C6—C9—N1	180.0
C9—C6—C7—C8	0.0	C7—C6—C9—C11	−115.91 (11)
O3—N1—C8—C7	180.0	C5—C6—C9—C11	64.09 (11)
C9—N1—C8—C7	0.0	C7—C6—C9—C11^i^	115.92 (11)
O3—N1—C8—C10^i^	−61.39 (10)	C5—C6—C9—C11^i^	−64.08 (11)

Symmetry codes: (i) *x*, −*y*, *z*; (ii) −*x*+2, −*y*, −*z*+1.

### Hydrogen-bond geometry (Å, °)

**Table:**

*D*—H···*A*	*D*—H	H···*A*	*D*···*A*	*D*—H···*A*
C11—H11C···C3^iii^	0.98	2.91	3.745 (2)	143

Symmetry codes: (iii) *x*−1, *y*, *z*.
